# Reasons Why People Change Their Alcohol Consumption in Later Life: Findings from the Whitehall II Cohort Study

**DOI:** 10.1371/journal.pone.0119421

**Published:** 2015-03-10

**Authors:** Annie Britton, Steven Bell

**Affiliations:** Research Department of Epidemiology and Public Health, University College London, London, United Kingdom; CUNY, UNITED STATES

## Abstract

**Purpose:**

Harmful alcohol consumption among the ageing population is an important public health issue. Very few studies ask drinkers why they change their consumption in later life. The aim of this paper was to determine whether a group of people aged over 60 years increased or decreased their alcohol consumption over the past decade and to determine the reasons for their change. We also examined whether the responses varied by age, sex and socio-economic position (SEP).

**Subjects and Methods:**

Data were taken from 6,011 participants (4,310 men, 1,701 women, age range 61 to 85 years) who completed questionnaires at phase 11 (2012-2013) of the Whitehall II Cohort Study.

**Results:**

Over half the study members reported a change in alcohol consumption over the past decade (40% decreased, 11% increased). The most common reasons given for decreases were as a health precaution and fewer social occasions. Common reasons for increases were more social occasions and fewer responsibilities. The lowest SEP group was less likely to increase consumption compared to high SEP (RR 0.57, 95% CI 0.40 to 0.81). Women were more likely to increase consumption in response to stress/depression than men (RR1.53, 95% CI 1.04 to 2.25). Compared to high SEP, the lowest SEP group was less likely to reduce as a health precaution (RR 0.61, 95% CI 0.38 to 0.76).

**Conclusions:**

Alcohol consumption in late life is not fixed. Reasons for change vary by age, sex and SEP. Such information could be used to tailor intervention strategies to reduce harmful consumption.

## Introduction

Alcohol and ageing is emerging as an important public health issue.[1,2] There are concerns that drinking among the elderly may become a hidden epidemic and that older people with substance use problems have high levels of unmet need.[3,4] This section of society has more co-morbidity, is more likely to be taking prescribed medication[5] and more physiologically vulnerable to the effects of alcohol.[1] Older people can and do benefit from treatment and in some cases have better outcomes than younger people.[3]

We know that individuals change their alcohol consumption over the life-course and that mean volume consumed typically declines as people get older.[6] [7] Drinking motives have been fairly extensively studied among adolescents and young adults.[8,9] However, there has been little research on the reasons why people reduce or increase their consumption in later life. Such information may be used to inform future, targeted prevention programmes.[10]

The aim of this paper was to determine whether a group of people aged over 60 years increased or decreased their alcohol consumption over the past decade and to clarify the reasons for their change. We also examined whether the responses varied by age, sex and socio-economic position (SEP).

## Materials and Methods

The Whitehall II study is an ongoing cohort study of men and women, originally employed by the British civil service. The target population was all London-based office staff, aged 35–55 years. A total of 10,308 persons (6,895 men and 3,413 women), response rate 74%, were recruited to the study over 1985 to 1988.[11] Participants have been followed up regularly through a combination of clinical examinations and self-administered questionnaires covering demographic, health, work and lifestyle characteristics. The current data are from 6,011 participants (4,310 men, 1,701 women) who took part at phase 11 (2012–2013). This is 79% of those still alive, not withdrawn or lost. They ranged in age from 61 to 85 years. Participants provided written informed consent to participate in the study and the University College London Medical School Committee on the ethics of human research approved the study.

All participants were asked in a self-administered questionnaire whether they had taken an alcoholic drink in the past 12 months (responses yes or no). If they had not had a drink they were asked if they had always been a non-drinker. Participants who were not life-long non-drinkers (95.7% of participants) were then asked “Have you given up or reduced your alcohol consumption in the past 10 years? (responses yes or no) If yes, what were the main reasons?” They were given the following seven options and asked to tick all that apply: Illness/medication; health precaution/to prevent illness; I’ve had alcohol problems in the past; Pressure/concern from family/friends; To save money; Fewer social occasions involving alcohol consumption; Other (please specify).

Drinkers were also asked “Have you increased your alcohol consumption in the past 10 years? (responses yes or no) If yes, what were the main reasons?” They were given the following seven options and asked to tick all that apply: More social occasions involving alcohol; less responsibilities; bereavement/loneliness; to get to sleep; to relieve pain; to reduce stress/anxiety/depression; Other (please specify).

Where participants specified a reason in the “other” section, their responses were transcribed and coded.

At every phase of the study, participants were asked their civil service grade. This was used as an indicator of socio-economic position. If participants were no longer working in the civil service or had retired, their last known grade was used.

Modified Poisson regression was used to estimate relative risks (RR) and their associated 95% confidence intervals (CI) for changes in consumption as well as the reasons for change.[12] Separate models were fitted for each option and adjustment was made for age, gender and SEP. All models were estimated using Stata 13.1.[13]

## Results

Table 1 shows the characteristics of the participants at phase 11 of the study. Fifty-six percent were aged 60–69 years and 44.4% were aged 70 to 85 years. There were fewer people in lowest SEP group, especially among the men (only 3.7%). 93.6% had consumed an alcoholic drink in the past 12 months. Only 259 participants reported to be always a non-drinker. Just over 40% reported that they had reduced their consumption in the previous ten years (40.0% men, 41.2% women) and nearly 11% reported that they had increased their consumption (11.7% men, 8.9% women).

**Table 1:** Characteristics of participants at phase 11 of the Whitehall II study.

n (%)	Men	Women	Total
Age: 60–69 years	2471 (57.3)	917 (53.9)	3388 (56.4)
70+ years	1839 (42.7)	784 (46.1)	2623 (43.6)
Socio-economic position: High	2490 (57.8)	447 (26.3)	2937 (48.9)
Intermediate	1667 (38.7)	853 (50.1)	2520 (41.9)
Low	153 (3.5)	401 (23.6)	554 (9.2)
Alcoholic drink in past 12 months?	4120 (95.6)	1506 (88.5)	5626 (93.6)
Always a non-drinker?	114 (2.6)	145 (8.5)	259 (4.3)
Reduced consumption in past 10 years?	1716 (40.0)	686 (41.2)	2402 (40.3)
Increased consumption in past 10 years?	502 (11.7)	149 (8.9)	651 (10.9)

The reasons for reductions are shown in Table 2. The most common reasons for reductions were as a health precaution (45% men, 34% women) and fewer social occasions (46% men, 41% women). 720 people gave “other” reasons for reductions. Common responses included no desire to drink (n = 209), worried about the consequences (n = 109), attempting to control weight (n = 96), and more driving (n = 45) (data not shown).

**Table 2:** Cross-tabulation of reasons for reductions (n = 2402) and reasons for increases (n = 651) in alcohol consumption in the previous 10 years.

Reasons for reductions (n (%))							
	Illness/ medication	Health precaution	Alcohol problems in the past	Pressure from family/ friends	To save money	Fewer social occasions	Other
Men (n = 1716)	355 (20.7)	767 (44.7)	37 (2.2)	72 (4.2)	196 (11.4)	783 (45.6)	475 (27.7)
Women (n = 686)	150 (21.9)	232 (33.8)	10 (1.5)	8 (1.2)	41 (6.0)	284 (41.4)	245 (35.7)
60–69 (n = 1387)	236 (17.0)	612 (44.1)	36 (2.6)	58 (4.2)	133 (9.6)	606 (43.7)	424 (30.6)
70+ (n = 1015)	269 (26.5)	387 (38.1)	11 (1.1)	23 (2.2)	104 (10.2)	461 (45.4)	296 (29.2)
Socio-economic position: High (n = 1156)	201 (17.4)	532 (46.0)	16 (1.4)	38 (3.3)	78 (6.7)	482 (41.7)	383 (33.1)
Intermediate (n = 1019)	251 (24.6)	410 (40.2)	27 (2.6)	37 (3.6)	140 (13.7)	486 (47.7)	278 (27.3)
Low (n = 227)	53 (23.3)	57 (25.1)	4 (1.8)	5 (2.2)	19 (8.4)	99 (43.6)	59 (26.0)

Reasons for increases (n (%))							
	More social occasions	Less responsibilities	Bereavement/ loneliness	To get to sleep	To relieve pain	To reduce stress	other
Men (n = 502)	252 (50.2)	185 (36.9)	38 (7.6)	29 (5.8)	11 (2.2)	70 (13.9)	189 (37.6)
Women (n = 149)	78 (52.3)	40 (26.8)	15 (10.1)	8 (5.4)	3 (2.0)	35 (23.5)	51 (34.2)
60–69 (n = 420)	221 (52.6)	169 (40.2)	27 (6.4)	24 (5.7)	9 (2.1)	64 (15.2)	153 (36.4)
70+ (n = 231)	109 (47.2)	56 (24.2)	26 (11.3)	13 (5.6)	5 (2.2)	41 (17.7)	87 (37.7)
Socio-economic position: High (n = 364)	178 (48.9)	139 (38.2)	24 (6.6)	16 (4.4)	8 (2.2)	47 (12.9)	147 (40.4)
Intermediate (n = 253)	131 (51.8)	77 (30.4)	27 (6.7)	17 (6.7)	4 (1.6)	50 (19.8)	85 (33.6)
Low (n = 34)	21 (61.8)	9 (26.5)	2 (5.9)	4 (11.8)	2 (5.9)	8 (23.5)	8 (23.5)

The most common reasons for increases (Table 2) were more social occasions (50% men, 52% women), less responsibilities (37% men, 27% women). 240 gave “other” reasons for increases. Common responses included pleasure (n = 67), retirement (n = 34), drinking with meals (n = 43), habit (n = 13) and affordability (n = 12) (data not shown).

The regression estimates for sex, age and SEP are shown in Table 3. Older people were more likely to reduce due to illness (≥70 years vs. 60–69 years RR 1.55, 95% CI 1.33 to 1.81). Compared to high SEP, low SEP group was less likely to reduce as a health precaution (RR 0.61, 95% CI 0.48 to 0.76). Older people and women were less likely to reduce due to concern from their family (≥70 years vs. 60–69 years RR 0.53, 95% CI 0.33 to 0.86; Women vs. men RR 0.27, 95% CI 0.13 to 0.56). Lower SEP groups were more likely to reduce as a means of saving money, whilst women were less likely than men. (Low SEP vs. high SEP RR 1.84, 95% CI 1.06 to 3.18; women vs. men RR 0.42 95% CI 0.29 to 0.61). Women were less likely than men to cite fewer social occasions as a reason for reduction (RR 0.80, 95% CI 0.66 to 0.97).

**Table 3:** Relative risks [95% Confidence Intervals] for changes in alcohol consumption in the past 10 years and reasons for doing so by sex, age and socio-economic position.

Reasons for reductions							
	Reduced	Illness/ medication	Health precaution	Alcohol problems in the past	Pressure from family/ friends	To save money	Fewer social occasions
Men (n = 1716)	1.00	1.00	1.00	1.00	1.00	1.00	1.00
Women (n = 686)	1.01 [0.94,1.09]	0.98 [0.83,1.17]	0.82** [0.73,0.93]	0.61 [0.30,1.24]	0.27*** [0.13,0.56]	0.46*** [0.33,0.65]	0.88* [0.79,0.98]
60–69 years (n = 1387)	1.00	1.00	1.00	1.00	1.00	1.00	1.00
70+ years (n = 1015)	0.95 [0.89,1.01]	1.55*** [1.33,1.81]	0.89* [0.80,0.98]	0.42* [0.21,0.81]	0.53** [0.33,0.86]	1.08 [0.85,1.38]	1.04 [0.95,1.14]
Socio-economic Position: High (n = 1156)	1.00	1.00	1.00	1.00	1.00	1.00	1.00
Intermediate (n = 1019)	1.03 [0.96,1.10]	1.42*** [1.20,1.67]	0.90* [0.81,0.99]	2.02* [1.10,3.71]	1.24 [0.79,1.92]	2.21*** [1.70,2.88]	1.16** [1.06,1.28]
Low (n = 227)	1.10 [0.98,1.23]	1.25 [0.94,1.66]	0.61*** [0.48,0.78]	1.82 [0.60,5.48]	1.22 [0.48,3.05]	1.73* [1.05,2.86]	1.10 [0.93,1.31]

Reasons for increases							
	Increased	More social occasions	Less responsibilities	Bereavement/ loneliness	To get to sleep	To relieve pain	To reduce stress
Men (n = 502)	1.00	1.00	1.00	1.00	1.00	1.00	1.00
Women (n = 149)	0.86 [0.71,1.03]	1.00 [0.83,1.21]	0.78 [0.58,1.04]	1.26 [0.67,2.39]	0.70 [0.28,1.79]	0.69 [0.21,2.31]	1.53* [1.04,2.25]
60–69 years (n = 420)	1.00	1.00	1.00	1.00	1.00	1.00	1.00
70+ years (n = 231)	0.72*** [0.62,0.84]	0.89 [0.76,1.05]	0.61*** [0.47,0.79]	1.72* [1.02,2.90]	0.99 [0.52,1.89]	1.02 [0.34,3.05]	1.13 [0.79,1.61]
Socio-economic Position: High (n = 364)	1.00	1.00	1.00	1.00	1.00	1.00	1.00
Intermediate (n = 253)	0.83* [0.71,0.97]	1.06 [0.90,1.25]	0.83 [0.66,1.04]	1.53 [0.8882.67]	1.60 [0.81,3.14]	0.75 [0.22,2.59]	1.43 [0.98,2.07]
Low (n = 34)	0.57** [0.40,0.81]	1.27 [0.93,1.73]	0.81 [0.45,1.46]	0.75 [0.16,3.51]	3.28 [0.93,11.61]	3.32 [0.78,14.09]	1.40 [0.70,2.80]

All models adjusted for sex, age and socioeconomic position.

* p<0.05,

** p<0.01,

*** p<0.001.

The lowest SEP group was less likely to increase consumption when compared to the high SEP (RR 0.57, 95% CI 0.40 to 0.81). Older people were less likely to report less responsibilities as a reason for increasing consumption (RR 0.61, 95% CI 0.47 to 0.79). Older people were more likely to increase drinking in response to bereavements/loneliness (RR 1.72, 95% CI 1.02 to 2.90). Women were more likely to increase consumption in response to stress/depression than men (RR 1.53, 95% CI 1.04 to 2.25).

## Discussion

In our study of 6,011 elderly men and women, we found that over half the study members reported that they had changed their alcohol consumption over the past decade (40% decreased, 11% increased). The most common reasons given for decreases were as a health precaution and fewer social occasions. Conversely, common reasons for increases were more social occasions and fewer responsibilities. We noted significant differences in response by age, sex, age and SEP.

Previous studies have documented various risk factors for increases in consumption in later life, for example, more time and opportunity to drink, and pain reduction and insomnia.[14] However, there have been few studies which ask older people’s own reasoning for their alcohol consumption. Immonen and colleagues investigated what older Finnish adults (aged 65 years and above) considered to be the reasons for their drinking [15]. The most common reasons were “having fun or celebration” (58.7%), “for social reason” (54.2%) and “for medicinal purposes” (20.1%). The latter finding is particularly of interest. In our study medicinal benefits of alcohol was not offered as an option for increasing consumption. When coding the “other” option, only eight participants in the Whitehall II cohort reported that they had increased consumption as they believed alcohol was good for their health. This is clearly much lower than the Finnish proportion (although we cannot rule out that many of the Whitehall II participants are drinking for believed health benefits, they just have not increased consumption over last decade). In a small study in New Zealand, 141 people aged over 65 years were asked for reasons for changing their consumption over the past 12 months[16]. Among the nine who increased their consumption, the reasons were encouragement from friends, loneliness, as an alternative to smoking and enjoyment of drinking alcohol. Among the 43 who decreased their drinking, the main reasons were for health concerns or because of pressure from family and friends. These findings are broadly in line with those from our larger study.

Reassuringly, in our study, few respondents increased consumption as a strategy to relieve pain or insomnia. However, stress reduction was a common reason and this could be a potential intervention opportunity. Informing people of other ways to cope with stress, and bereavement and loneliness may reduce unhealthy drinking habits[17].

Our finding that lower SEP groups were less likely than the high SEP group to increase their consumption supports concerns about increases in consumption amongst older affluent drinkers[3]. This may lead to a rise in alcohol related harm in higher SEP groups and therefore this information needs to be relayed to primary care workers who may detect alcohol misuse. We also found that those in lower SEP groups were less likely to cite health precaution as a reason to reduce consumption. This could possibly contribute to social inequalities in health in later life.

To our knowledge this is the largest study in which drinkers were asked the reasons for changes in alcohol consumption. However, the sample was based on British civil servants who may not be representative of all drinkers within the population. Very heavy drinkers are under-represented in population surveys [18]. By phase 11, about 28 years after baseline, the remaining sample was 6,011 which is 79% of those still alive and not withdrawn or lost. It is likely that those remaining in the sample are a healthier subsample [19] [20]). Furthermore, we did not ask participants how much they had changed their consumption, but left it to their own interpretation of ‘increase’ or ‘decrease’. Future work should look at changing patterns of consumption in later life which may have different health consequences [21]. Offering a selection of reasons is likely to have influenced participants’ responses, however we also included a free text box for “other” reasons.

Nevertheless, this study provides insights on the motives for why people change consumption in later life by actually asking the reasons, rather than determining risk factors associated with change, as has more commonly been done. This information has the potential to be used to help tailor targeted intervention programmes to reduce harmful alcohol consumption in later life. In particular, the significant differences in reasons for change by age, sex and SEP that we detected in our study could be useful for detection of alcohol misuse in later life.
